# Understanding the Positive Associations of Sleep, Physical Activity, Fruit and Vegetable Intake as Predictors of Quality of Life and Subjective Health Across Age Groups: A Theory Based, Cross-Sectional Web-Based Study

**DOI:** 10.3389/fpsyg.2018.00977

**Published:** 2018-06-18

**Authors:** Shu Ling Tan, Vera Storm, Dominique A. Reinwand, Julian Wienert, Hein de Vries, Sonia Lippke

**Affiliations:** ^1^Health Psychology and Behavioral Medicine, Department of Psychology and Methods, Jacobs University Bremen, Bremen, Germany; ^2^Institute of Sport and Exercise Sciences, Department of Social Sciences of Sport, University of Münster, Münster, Germany; ^3^Institute of Sport and Exercise Sciences, Department of Sport Psychology, University of Münster, Münster, Germany; ^4^Rehabilitative Gerontology, Department of Special Education and Rehabilitation, Faculty of Human Sciences, University of Cologne, Cologne, Germany; ^5^Scientific Institute of TK for Benefit and Efficiency in Health Care (WINEG), Hamburg, Germany; ^6^Department of Health Promotion, CAPHRI School for Public Health and Primary Care, Maastricht University, Maastricht, Netherlands; ^7^Bremen International Graduate School of Social Sciences, Jacobs University Bremen, Bremen, Germany

**Keywords:** sleep, physical activity, fruit and vegetable intake, sleep quality, quality of life, subjective health, multiple health behaviors, age-group differences

## Abstract

**Background:** Due to the increase in unhealthy lifestyles and associated health risks, the promotion of healthy lifestyles to improve the prevention of non-communicable diseases is imperative. Thus, research aiming to identify strategies to modify health behaviors has been encouraged. Little is known about addressing multiple health behaviors across age groups (i.e., young, middle-aged, and older adults) and the underlying mechanisms. The theoretical framework of this study is Compensatory Carry-Over Action Model which postulates that different health behaviors (i.e., physical activity and fruit and vegetable intake) are interrelated, and they are driven by underlying mechanisms (more details in the main text). Additionally, restful sleep as one of the main indicators of good sleep quality has been suggested as a mechanism that relates to other health behaviors and well-being, and should therefore also be investigated within this study. The present study aims to identify the interrelations of restful sleep, physical activity, fruit and vegetable intake, and their associations with sleep quality as well as overall quality of life and subjective health in different age groups.

**Methods:** A web-based cross-sectional study was conducted in Germany and the Netherlands. 790 participants aged 20–85 years filled in the web-based baseline questionnaire about their restful sleep, physical activity, fruit and vegetable intake, sleep quality, quality of life, and subjective health. Descriptive analysis, multivariate analysis of covariance, path analysis, and multi-group analysis were conducted.

**Results:** Restful sleep, physical activity, and fruit and vegetable intake were associated with increased sleep quality, which in turn was associated with increased overall quality of life and subjective health. The path analysis model fitted the data well, and there were age-group differences regarding multiple health behaviors and sleep quality, quality of life, and subjective health. Compared to young and older adults, middle-aged adults showed poorest sleep quality and overall quality of life and subjective health, which were associated with less engagement in multiple health behaviors.

**Conclusion:** A better understanding of age-group differences in clustering of health behaviors may set the stage for designing effective customized age-specific interventions to improve health and well-being in general and clinical settings.

**Trial Registration:** A clinical trial registration was conducted with ClinicalTrials.gov (NCT01909349) https://clinicaltrials.gov/ct2/show/NCT01909349.

## Introduction

Non-communicable diseases, and especially cardiovascular diseases (CVDs) account for most premature deaths globally, followed by cancers, respiratory diseases, diabetes, and obesity (World Health Organization [WHO], 2014). While an unhealthy lifestyle like physical inactivity and an unhealthy diet are the most common behaviors that increase the incidence of NCDs (Lachat et al., 2013; World Health Organization [WHO], 2014), sleep problems, such as inadequate sleep duration and poor sleep quality are also becoming more common and recognizable as unhealthy since numerous studies showed their associations with NCDs, especially, hypertension, type 2 diabetes, and obesity (Shan et al., 2015; Cassidy et al., 2016; Ferranti et al., 2016; Montag et al., 2017). Accordingly, a systematic review revealed that sleep problems should be mentioned during the visit to healthcare providers, as sleep problems may severely influence recovery from the diseases, control of NCDs, and may affect individuals’ quality of life in general (Surani et al., 2015).

Sleep has gained attention in research and it has been strongly suggested to raise awareness of sleep as an important health behavior (Perry et al., 2013; Chaput and Dutil, 2016). In particular, good quality and restful sleep not only reduce health risks with the chances for recovery and rejuvenation (Eek et al., 2012; Barber, 2014), but also might facilitate quality of life by enhancing well-being (Palmer and Alfano, 2017).

A healthy lifestyle contains more than single health behaviors, with the balance of energy expenditure (i.e., physical activity) and energy intake (i.e., healthy food intake), and the success in one behavior could facilitate changes in other behaviors (Lippke, 2014; Geller et al., 2017). In fact, physical activity and fruit and vegetable intake are two of the most important and easiest to influence health behaviors which effectively reduce the risks of NCDs (Lachat et al., 2013; World Health Organization [WHO], 2014), while physical activity and fruit and vegetable intake were positively interrelated within a multiple health behavior study (Fleig et al., 2015). Evidently, physical activity and fruit and vegetable intake are associated with lower mortality (Tian et al., 2017), higher self-rated health (Södergren et al., 2012), and better health-related quality of life (Kwon et al., 2015).

In addition, many previous studies found that different behaviors are related. For example, inadequate sleep duration and poor sleep quality are associated with unhealthy diet and physical inactivity (Grandner et al., 2015; Kittle et al., 2016). A study showed that sufficient sleep and good sleep quality were positively associated with a higher intake of dietary fiber and fruit and vegetable consumption (Ferranti et al., 2016). Furthermore, a systematic review identified that sleep and diet facilitate each other with carry-over mechanisms (Frank et al., 2017), including positive experiences, skills, cognitions like motivation, positive emotion, and self-regulatory strategies, and thus have impacts throughout the life course (Lippke, 2014). However, inadequate sleep duration also changed circadian rhythms and hormonal levels and contributed to obesity, diabetes, and CVDs (Frank et al., 2017). An online survey study in a web-based platform found that individuals who had more physical activity, fruit and vegetable intake, and slept well were more likely to report better mood (Liu et al., 2012). Therefore, another study strongly argued that sleep should also be taken into consideration for health improvements, and given as much attention as physical activity and fruit and vegetable intake as it seems reasonable that these multiple health behaviors influence overall health (Chaput and Dutil, 2016). However, the interrelations of physical activity and fruit and vegetable intake with sleep are lacking. Consequently, it is worthwhile to examine the interrelations of these health behaviors altogether.

With increasing age, the number of people suffering from NCDs also increases. It is known that most premature deaths attributed to NCDs occur between the ages of 30 and 70 years, and thus, NCDs most likely affect people of all age groups (World Health Organization [WHO], 2014, 2017). However, motivation to and success in health behavior change differs by age and subjective health with distinct health perceptions and habits (Deeks et al., 2009), while different age groups might yield noticeably different approaches in improving health. A recent study showed that, in comparison to younger adults, older adults were more likely to meet fruit and vegetable recommendations but were less likely to meet the physical activity recommendations (Alley et al., 2017).

Moreover, a study reported that the predictors of sleep quality differ between young adults and older adults: sleep duration was shorter with increased age, and older adults reported less restful sleep than younger adults (Zilli et al., 2009). Previous studies revealed that differences in health behaviors and sleep quality can be documented across age groups. For instance, a good sleep was positively associated with fruit and vegetable intake among young adolescents (Ferranti et al., 2016). Additionally, middle-aged and older adults reported poor sleep quality with low levels of physical activity and therefore yielded poorest health characteristics (Rayward et al., 2017). Moreover, a previous study asked people about their perceptions of sleep quality and they defined one of the main indicators of good sleep quality is feeling rested with few or no sleep disruption during the night (Harvey et al., 2008). Sleep problems such as unrestful sleep increase with age. Thus, there is a growing interest in understanding the role of restful sleep to many important health outcomes beyond sleep duration across age groups (Kittle et al., 2016).

A healthy lifestyle should be initiated earlier throughout young adulthood as it is related to lower risks of CVDs in middle age and later life (Liu et al., 2012). While sleep might be one of the body’s most important mechanisms, little about it has been studied in relation to other health behaviors. Consequently, more scientific evidence of age-group differences among these multiple health behaviors is needed. Thus, this present study examined underlying behavioral and psychological mechanisms across age groups. Age groups should be analyzed in regard to whether young adults (i.e., 20–35 years) differ from middle-aged adults (i.e., 36–55 years) and the aging older adults that included both young-old- and oldest-old adults (i.e., older than 56 years). This grouping approach was chosen by previous study related to health behavior change (Petry, 2002; Ziegelmann et al., 2006), and some studies which are related to the aspects of aging (Palmiero et al., 2017; Wang et al., 2017).

Looking at the associations with underlying mechanisms, sleep has been found to be associated with physical activity and fruit and vegetable intake with increased life satisfaction and perceived health, as well as decreased high blood pressure among participants who were employees and mostly middle-aged (Merrill et al., 2011). Within this study, the outcomes highlighted that the multiple health behavior change interventions were effective and showed significant improvements among middle-aged employees with self-rated better health. Compared to older adults who reported having good sleep quality, older adults with poor sleep quality rated poorer subjective health and more frequently experienced restless sleep (Abraham et al., 2017). Among young adults, sleep problems were associated with lower quality of life and subjective health (Chen et al., 2014), but physical activity was not related to better quality of life (Häyrinen and Tarkka, 2016). Poor sleep was related to a decreased likelihood of maintaining healthy behavior change (Hui and Grandner, 2015), and it is thus essential to examine sleep quality and restful sleep further to provide more information on how sleep improvement, together with physical activity and fruit and vegetable intake could be considered.

Most theoretical frameworks describe only single behaviors, for example, the Health Action Process Approach (HAPA, Schwarzer, 2008; Schwarzer et al., 2011) describes single health behavior change of adoption, initiation, and maintenance of health behavior via motivation and volition phases, including factors such as intention, self-efficacy, outcome expectancies, risk perceptions, and action planning. However, to promote a healthy lifestyle and to prevent NCDs, it is necessary to take multiple health behaviors into account (Prochaska et al., 2010). Like the CCAM originated by Lippke (2014) is a novel approach for understanding the multiple health behavior change process with the underlying mechanisms that promote a healthy lifestyle and prevent NCDs. The CCAM addresses multiple health behaviors via carry-over mechanisms, and their associations with social-cognitive factors, such as intention, planning, and self-efficacy. Although these social-cognitive factors are essential in coping with tempting situations and compensatory cognitions that occur during the process of behavior change, these factors are not the purposes of this study. The research questions of this study are driven by the assumptions of the CCAM which can be studied individually: (1) different health behaviors interrelate, in this case, restful sleep, physical activity, and fruit and vegetable intake interrelate with carry-over mechanisms; (2) a healthy lifestyle contains more than a single health behavior, which decreases the stress reaction while it increases an individuals’ well-being (in this case, sleep quality), and associates with higher-level goal (in this case, quality of life and subjective health).

People experience different kinds of stressors while adopting and maintaining healthy behaviors. For example, if a person has set a goal and fails to reach this goal (e.g., due to work stress, daily hassles, or chronic stressors), their well-being might be affected. This is especially the case for work stress which has been identified as one of the main risk factors for hypertension and CVDs among young adults (Mucci et al., 2016). Thus, good sleep quality may result in the decrease of the stress reaction and the increase of well-being. This is especially true when sleep has been portrayed as an approach for achieving a sufficient recovery state to reenergize for future tasks or cope with chronic diseases, and for revitalizing self-control, which increases self-regulatory behavior and consequently decreasing stress symptoms (Barber, 2014).

Engagements in multiple health behaviors may lead to increased well-being (Geller et al., 2017), thus, many studies examined the associations between health behaviors and underlying behavioral and psychological mechanisms (de Vries et al., 2008; Prochaska et al., 2010; Schulz et al., 2014; Lippke et al., 2015; Reinwand et al., 2016; Storm et al., 2016; Duan et al., 2017). For example, a study examined students’ health and well-being and found that low level of engagement in health-promoting lifestyle-related behaviors, such as physical activity and nutrition, and stress management were associated with poor sleep quality and poor subjective health (Araújo et al., 2017). Nonetheless, little is known about the interrelations of restful sleep, physical activity, and fruit and vegetable intake, and their associations with sleep quality, quality of life, and subjective health, for the general population and participants that were motivated to decrease their risk for a CVD. Therefore, based on the CCAM, the current study aims:

(1) To identify the interrelations among restful sleep, physical activity, fruit and vegetable intake, sleep quality, quality of life, and subjective health.
(2) To examine to what extent restful sleep, physical activity, and fruit and vegetable intake are associated with increased sleep quality, quality of life, and subjective health.
(3) To investigate age-group differences in health behaviors (i.e., restful sleep, physical activity, and fruit and vegetable intake), and sleep quality, quality of life, and subjective health among young adults, middle-aged adults, and older adults.
(4) To understand the associations between the health behaviors (i.e., restful sleep, physical activity, and fruit and vegetable intake), and underlying mechanisms (i.e., sleep quality, quality of life, and subjective health) across age groups.

## Materials and Methods

### Study Design, Settings, Participants, and Procedures

This study is a cross-sectional study dataset, which stems from a randomized control trial (RCT) initially, to investigate whether a web-based, computer-tailored intervention is effective in increasing self-reported physical activity and fruit and vegetable intake. The results on the effectiveness of the intervention have been published elsewhere (Reinwand et al., 2015; Storm et al., 2016; Duan et al., 2017), and are distinct from the present study. Only the cross-sectional data from the self-report baseline questionnaires have been used to answer the research questions of this current study. The baseline questionnaire was the same for the intervention and waiting control group; thus, we expect no differences in results due to group condition.

A study protocol with detailed information of the study has been previously published (Reinwand et al., 2013). Thus, only a summary of the study methodology is mentioned in the following. This study has received ethical approval from the German Society for Psychology (Deutsche Gesellschaft für Psychologie, DGPs; EK-A-SL 022013) in Germany, and the Medical Ethics Committee of Atrium Medical Centre Heerlen (METC; 12-N-124) in the Netherlands. A clinical trial registration was conducted with ClinicalTrials.gov (Identifier: NCT01909349). All participants in this study gave informed consent online and obtained the access to the web-based program and the questionnaires. All participants participated voluntarily, and the data were anonymized.

A total of 1,010 participants were initially recruited from cardiac rehabilitation facilities and heart training groups, as well as Internet platforms and online panels in Germany and the Netherlands from 2013 to 2015. Only participants who met the following inclusion criteria were approached to take part in this study: (a) aged 20 years and older; (b) German or Dutch language proficiency; (c) having an interest to reduce cardiac risk behavior in terms of being able to be physically active at least 150 min per week and being able to eat at least five portions of fruit and vegetables a day; (d) no complications and restrictions for physical activity and fruit and vegetable intake; and (e) having Internet access.

The research team excluded a total of 220 datasets because of double registration (*n* = 5), non-available dataset (*n* = 128), inadequate age (*n* = 1 younger than 20 years), and missing gender information (*n* = 86). The final sample size is 790 participants.

### Measurement Instruments

#### Sociodemographic Variables

All socio-demographic characteristics were self-reported by the participants: year of birth, gender, country, employment status, marital status, and education levels in the baseline questionnaire. Based on participant’s information about body height and body weight, the BMI was calculated. The age of the participants is categorized into three age groups—young adults (1 = aged 20–35 years), middle-aged adults (2 = aged 36–55 years), and older adults (3 = aged 56 years and older). This categorization of age groups was adopted from the previous studies which investigated various relevant topics including health behavior change and aging aspects (Petry, 2002; Palmiero et al., 2017; Wang et al., 2017).

#### Health Behaviors: Physical Activity and Fruit and Vegetable Intake

To provide reliable individual health information from the web-based study, the assessment of participants’ physical activity and fruit and vegetable intake was done with the items “During the last weeks did you engage in physical activity at least 5 days a week for 30 min or more, in such a way that you were moderately exhausted?” and “During the last weeks, did you eat five portions of fruit and vegetables per day?” The answers were based on a 5-point Likert scale ranging from 1 = No, and I do not intend to do so; 2 = No, but I’m thinking about it; 3 = No, but I intend to do so; 4 = Yes, for a short period of time to 5 = Yes, for a long period of time. These items access the stages of change of health behaviors which combine intention and action in ordered categorical form, based on theoretical assumptions of the Transtheoretical Model (Prochaska and DiClemente, 1983) and HAPA model. The reliability and validity of these measures were found in these previous studies (Lippke et al., 2009).

#### Restful Sleep

With one of the items from the Center for Epidemiologic Studies Short Depression Scale (CES-D10) (Eaton et al., 2004), participants were asked to assess their level of restful sleep: “In the past week, my sleep was restless,” on a scale from 1 = rarely or none of the time (<1 day) to 4 = most or all the time (5–7 days) (Andresen et al., 1994). As this present study focuses on the positive aspects of sleep, this item has been reverse coded to measure restful sleep. This item has been included individually in the previous health-related studies (Kutner et al., 2001; El Ansari et al., 2011; Bassett and Moore, 2014).

#### Sleep Quality

To determine sleep quality, one item from the short version of the World Health Organization Quality of Life (WHOQOL-BREF) Questionnaire (Group WHOQOL, 1993; Skevington et al., 2004; Trompenaars et al., 2005; Hsiao et al., 2014) was used. The participants rated the item “How satisfied are you with your sleep?” on a scale from 1 = very dissatisfied to 5 = very satisfied. This item measures sleep quality for the physical health domain, but it has been examined individually in a previous study (Lu et al., 2011).

#### Quality of Life and Subjective Health

This web-based study included two items to assess the overall quality of life and subjective health which were taken from the WHOQOL-BREF. The participants were asked to answer the item “Please keep in mind your standards, hopes, pleasures, and outcomes, in the last 4 weeks, how would you rate your quality of life?” on a scale from 1 = very poor to 5 = very good and “How satisfied are you with your health?” on a scale from 1 = very dissatisfied to 5 = very satisfied (Group WHOQOL, 1993; Skevington et al., 2004; Trompenaars et al., 2005; Lu et al., 2011; Hsiao et al., 2014). Based on a recent review, well-being interrelates with subjective health and quality of life (Diener et al., 2017). Thus, these two items of quality of life and subjective health, with Pearson’s *r* = 0.50, *p* < 0.001, were combined into one index to measure participants’ aggregated quality of life and subjective health overall (Diener et al., 2017). This was also done in previous research (Johnson et al., 2016).

### Statistical Analysis

All statistical analyses were performed using IBM SPSS 24 and AMOS 24. In the preliminary analysis which described the key characteristics of the main study variables which are important for further analysis, chi-square tests were performed to detect differences in the categorical variables gender and country among the three age groups. Descriptive statistics and bivariate correlation determined the interrelations among the main continuous variables: physical activity, fruit and vegetable intake, restful sleep, sleep quality, quality of life and subjective health, age, and BMI. Pearson’s *r-*values were used to measure the effect size of the relationships among variables, with the purpose to avoid incorrect inferences, bias results, and less precise estimates (Field, 2009).

In order to answer the research questions 2, 3, and 4, the main analyses contain three parts: (1) a path analysis was conducted to generate a proposed model based on the CCAM; (2) comparing age-group differences on the means of main study variables by using one-way multivariate analysis of covariance (MANCOVA); and (3) a multi-group analysis in structural equation modeling was conducted to examine the proposed model across age groups.

The analyses confirmed that multicollinearity did not affect any of the significant effects reported below, and that multivariate normality was assumed. Homogeneity of variances was not assumed for the dependent variables: sleep quality, *F*(2,787) = 5.41, *p* = 0.005; restful sleep, *F*(2,787) = 4.96, *p* = 0.007; quality of life and subjective health, *F*(2,787) = 5.16, *p* = 0.006; fruit and vegetable intake, *F*(2,787) = 20.11, *p* < 0.001; except physical activity, *F*(2,787) = 1.65, *p* = 0.194. Thus, the Games-Howell test was used as recommended if the homogeneity of the variances assumption is violated (Games and Howell, 1976; Jaccard et al., 1984).

Analysis of variance (ANOVA) was employed to analyze the variations of the dependent variables for each age group. Games-Howell *post hoc* test was selected for group comparisons as recommended (Games and Howell, 1976; Jaccard et al., 1984), and Robust Tests of Equality of Means (Welch) with asymptotically *F* distributed were used (Field, 2009). Polynomial contrast analyses were adopted to examine both linear and nonlinear trends (i.e., quadratic terms) and the trends were examined with weighted terms for unequal sample sizes for each age group. BMI, country of origin, gender, employment status, marital status, and education levels were included as control variables. The level of two-tailed statistical significance was set at *p* < 0.05. We used Sidak statistical measures to adjust for multiple testing.

## Results

### Sample Characteristics

A total of 790 participants with a mean age of 50.9 years. Among them, 62.9% of the participants were female, 50.1% were working full-time, and 66.3% of the participants were married or in a relationship. Middle-aged adults were the majority in both countries, with 49.1% in Germany, and 50.9% in the Netherlands. **Table 1** provides an overview of the sociodemographic variables in this study, except education levels due to more than one cell showing frequencies below 1, which may fail to detect a genuine effect (Field, 2009).

**Table 1:** Descriptive statistics on sociodemographic variables.

Categorical variables	Chi-square (χ^2^)	*p*	Young adults	Middle-aged adults	Older adults	Total
			(*n* = 102)	(*n* = 422)	(*n* = 266)	(*N* = 790)
			(20–35 years)	(36–55 years)	(56–84 years)	(20–84 years)
			*n* (%)	*n* (%)	*n* (%)	
**Country**	8.74	0.013				
German			34 (33.3)	207 (49.1)	130 (48.9)	371 (47.0)
Dutch			68 (66.7)	215 (50.9)	136 (51.1)	419 (53.0)
Gender	40.50	<0.001				
Male			17 (16.7)	142 (33.6)	134 (50.4)	293 (37.1)
Female			85 (83.3)	280 (66.4)	132 (49.6)	497 (62.9)
**Employment status**	273.12	<0.001				
Working full-time			44 (43.1)	271 (64.2)	81 (30.5)	396 (50.1)
Working part-time			28 (27.5)	101 (23.9)	44 (16.5)	173 (21.9)
Vocational training			11 (10.8)	3 (0.7)	1 (0.4)	15 (1.9)
Unemployed			10 (9.8)	22 (5.2)	17 (6.4)	49 (6.2)
Retired			3 (2.9)	6 (1.4)	106 (13.4)	115 (14.6)
Housewife/husband			6 (5.9)	19 (4.5)	17 (6.4)	42 (5.3)
**Marital status**	98.23	<0.001				
Single			24 (23.5)	38 (9.0)	16 (6.0)	78 (9.9)
Close relationship not living together			16 (15.7)	20 (4.7)	10 (3.8)	46 (5.8)
Close relationship and living together			25 (24.5)	36 (8.5)	15 (5.6)	76 (9.6)
Married			35 (34.3)	294 (69.7)	195 (73.3)	524 (66.3)
Divorced			2 (2.0)	28 (6.6)	24 (9.0)	54 (6.8)
Widowed			0 (0.0)	6 (1.4)	6 (2.3)	12 (1.5)

### Correlations of Main Variables

For continuous variables, inter-correlation analysis (Pearson’s *r*) was used to inspect the interrelations of the main study variables as displayed in **Table 2**. In correlational findings, main study variables were interrelated, except restful sleep only significantly correlated with sleep quality and overall quality of life and subjective health (*p* < 0.05).

**Table 2:** Means, standard deviations, and correlations of the major study variables (*N* = 790).

Variables	1	2	3	4	5	6	7
1. Sleep quality	–	–	–	–	–	–	–
2. Restful sleep	0.56^∗∗^	–	–	–	–	–	–
3. Quality of life and subjective health	0.49^∗∗^	0.30^∗∗^	–	–	–	–	–
4. Physical activity	0.21^∗∗^	0.02	0.18^∗∗^	–	–	–	–
5. Fruit and vegetable intake	0.10^∗∗^	-0.03	0.18^∗∗^	0.23^∗∗^	–	–	–
6. Age	-0.06	-0.06	-0.01	0.18^∗∗^	0.06	–	–
7. BMI (kg/m^2^)	-0.10^∗∗^	-0.04	-0.28^∗∗^	-0.08^∗^	-0.11^∗∗^	0.12^∗∗^	–
*M*	3.32	3.74	7.13	3.45	3.18	50.85	27.55
*SD*	1.01	0.53	1.42	1.12	1.18	12.15	5.41
Range	1.00–5.00	2.00–4.00	2.00–10.00	1.00–5.00	1.00–5.00	20–84	15.20–55.00

*M, mean; *SD*, standard deviation. ^∗∗^*p* < 0.01 and ^∗^*p* < 0.05, significance levels of correlations.*

### Path Analysis of a Proposed Model

**FIGURE 1:**
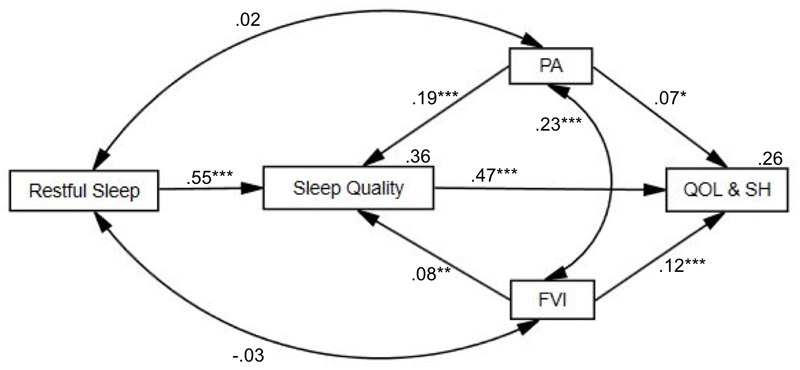
Conceptual overall path analysis model with standardized regression coefficients. The paths of restful sleep, physical activity (PA), fruit and vegetable intake (FVI), sleep quality, quality of life, and subjective health (QOL&SH). The examined variables accounted for 36% of the variance of sleep quality and 26% of the variance of quality of life and subjective health. ^∗∗∗^*p* < 0.001, ^∗∗^*p* < 0.01, and ^∗^*p* < 0.05.

To assess to what extent restful sleep, physical activity, and fruit and vegetable intake were associated with increased quality of life and subjective health, via sleep quality, a path analysis model was run, as displayed in **Figure 1**. Control variables like age and gender (dichotomous variable) were included as covariates to be able to understand the model as a whole. The overall model fitted the data well, with chi-square, χ^2^ (2, *N* = 790) = 3.77, *p* = 0.15, comparative fit index (CFI) = 0.998, Tucker-Lewis index (TLI) = 0.974, root mean-squared error of approximation (RMSEA) = 0.033.

Although restful sleep was not significantly interrelated with physical activity and fruit and vegetable intake, physical activity was positively interrelated with fruit and vegetable intake (β = 0.23, *B* = 0.30, *SE* = 0.05, *p* < 0.001). Restful sleep (β = 0.55, *B* = 1.04, *SE* = 0.06, *p* < 0.001), physical activity (β = 0.19, *B* = 0.17, *SE* = 0.03, *p* < 0.001), and fruit and vegetable intake (β = 0.08, *B* = 0.07, *SE* = 0.03, *p* = 0.011) were associated with increased sleep quality. Sleep quality was associated with increased quality of life and subjective health (β = 0.47, *B* = 0.66, *SE* = 0.04, *p* < 0.001). Further, physical activity and fruit and vegetable intake yielded positive associations with quality of life and subjective health, with β = 0.07, *B* = 0.09, *SE* = 0.04, *p* = 0.04, and β = 0.12, *B* = 0.15, *SE* = 0.04, *p* < 0.001, respectively. The examined variables accounted for 36% of the variance of sleep quality and 26% of the variance of quality of life and subjective health.

### Age Differences Among Multiple Health Behaviors and Underlying Mechanisms

The outcome of one-way MANCOVA yielded significant differences among age groups, with Wilks λ = 0.95, *F*(10,1554) = 3.831, *p* < 0.001, with partial eta squared, $\eta_{p}^{2}$ = 0.024. The *F*-statistics, *p-*values of significance levels, Games-Howell *post hoc* comparisons, linear and quadratic terms are displayed in **Table 3**. In quadratic trends, middle-aged adults yielded the lowest sleep quality, in which the mean is 3.20 and quality of life, and subjective health and the mean is 6.96. Physical activity showed significant differences in both linear and quadratic trends across age groups, in which older adults are having the most intention or action of being physically active and the mean is 3.70 and consuming fruit and vegetables with the mean 3.30.

**Table 3:** Analysis of age-group differences on main study variables.

	Total	Young adults	Middle-aged adults	Older adults	*F^a^* (*p*)	Games–Howell *post hoc* test	Linear term, *F*(*p*)	Quadratic term, *F*(*p*)
	(*N* = 790)	(*n* = 102)	(*n* = 422)	(*n* = 266)				
	*M* (*SE*)	*M* (*SE*)	*M* (*SE*)	*M* (*SE*)				
SQ	3.40 (0.42)	3.61 (0.10)	3.20 (0.05)	3.40 (0.07)	8.72 (0.00)	YA > MA < OA	0.77 (0.38)	17.49 (<0.001)
RS	3.75 (0.22)	3.79 (0.05)	3.74 (0.03)	3.73 (0.03)	1.99 (0.14)	–	3.07 (0.08)	0.04 (0.85)
QOL&SH	7.22 (0.06)	7.39 (0.14)	6.96 (0.07)	7.31 (0.07)	9.02 (0.00)	YA > MA < OA	0.16 (0.69)	17.83 (<0.001)
PA	3.45 (0.05)	3.31 (0.11)	3.32 (0.06)	3.70 (0.07)	18.56 (0.00)	YA < OA > MA	24.19 (<0.001)	12.45 (<0.001)
FVI	3.20 (0.05)	3.20 (0.12)	3.10 (0.06)	3.30 (0.08)	2.74 (0.07)	OA > MA	1.58 (0.21)	4.22 (0.04)

*SQ, sleep quality; RS, restful sleep; QOL&SH, overall quality of life and subjective health; PA, physical activity; FVI, fruit and vegetable intake; *M*, mean; *SE*, standard error; *N*, sample size; *F*^*a*^, asymptotically *F* distributed, Welch. For Games–Howell *post hoc* test, display with significance level at *p* < 0.05.*

### Path Analysis of Underlying Mechanisms Across Age Groups

**FIGURE 2:**
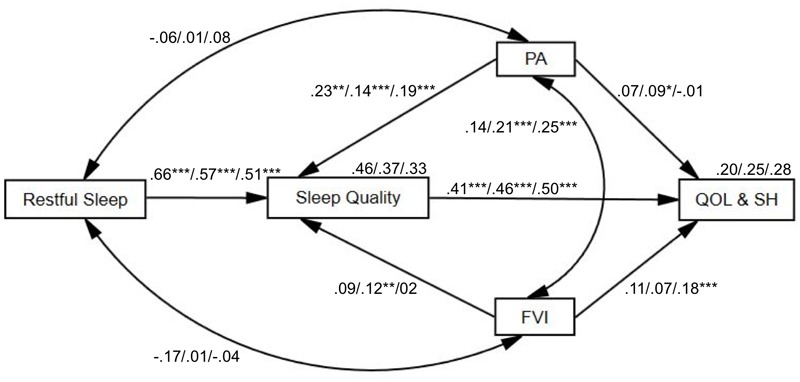
Conceptual path analysis model of age-group differences (young adults/middle-aged adults/older adults) with standardized regression coefficients. The paths of restful sleep, physical activity (PA), fruit and vegetable intake (FVI), sleep quality, and quality of life and subjective health (QOL&SH). The examined variables accounted for 46%/37%/33% of the variance of sleep quality and 20%/25%/28% of the variance of quality of life and subjective health accordingly to the age groups. ^∗∗∗^*p* < 0.001, ^∗∗^*p* < 0.01, and ^∗^*p* < 0.05.

To compare age-group differences across the main study variables, the *multi-group analysis* in structural equation modeling was performed. Gender (dichotomous variable) was included as the covariate, without including age since age differences are explored. The fully unconstrained path model provided an adequate fit to the data, with χ^2^ (3, *N* = 790) = 6.07, *p* = 0.11; CFI = 0.995, TLI = 0.926, RMSEA = 0.036. With model comparison, χ^2^ = 23.54; *df* = 16, *p* = 0.10, with *p* < 0.05 which could be the indications for differences among the age groups in the whole model, with the structural weights model fitted acceptably well too as χ^2^ (19, *N* = 790) = 29.62, *p* = 0.06; CFI = 0.983, TLI = 0.960, and RMSEA = 0.027 (**Figure 2**).

For young adults, restful sleep (β = 0.66, *B* = 1.71, *SE* = 0.19, *p* < 0.001) and physical activity (β = 0.23, *B* = 0.22, *SE* = 0.07, *p* = 0.002) were positively correlated with sleep quality. Sleep quality was associated with increased quality of life and subjective health (β = 0.41, *B* = 0.55, *SE* = 0.12, *p* < 0.001).

For middle-aged adults, restful sleep (β = 0.57, *B* = 0.99, *SE* = 0.07, *p* < 0.001), physical activity (β = 0.14, *B* = 0.12, *SE* = 0.03, *p* < 0.001), and fruit and vegetable intake (β = 0.12, *B* = 0.10, *SE* = 0.04, *p* = 0.003) were significantly positively associated with sleep quality. Sleep quality was associated with increased quality of life and subjective health (β = 0.46, *B* = 0.65, *SE* = 0.06, *p* < 0.001). Physical activity and fruit and vegetable intake were significantly interrelated with each other (β = 0.21, *B* = 0.24, *SE* = 0.06, *p* < 0.001). Physical activity showed significant positive association with quality of life and subjective health, with β = 0.09, *B* = 0.12, *SE* = 0.05, *p* = 0.03.

For older adults, restful sleep (β = 0.51, *B* = 0.995, *SE* = 0.10, *p* < 0.001) and physical activity (β = 0.19, *B* = 0.19, *SE* = 0.05, *p* < 0.001) were associated with sleep quality. Sleep quality was associated with quality of life and subjective health (β = 0.50, *B* = 0.70, *SE* = 0.08, *p* < 0.001). Physical activity and fruit and vegetable intake were interrelated with each other (β = 0.25, *B* = 0.36, *SE* = 0.09, *p* < 0.001). Further, fruit and vegetable intake was related with quality of life and subjective health (β = 0.18, *B* = 0.20, *SE* = 0.06, *p* = 0.001).

## Discussion

The purpose of this web-based study was to investigate age-group differences in the interrelations of multiple health behaviors (i.e., restful sleep, physical activity, and fruit and vegetable intake) and their associations with sleep quality, quality of life, and subjective health among participants who were motivated to reduce their cardiovascular risk.

### Summary of the Main Findings

To summarize, in the present study we could show there were interrelations between different health behaviors and that the strength of these interrelations differed among age groups. Regarding the first research question, we found that physical activity was associated with fruit and vegetable intake but neither of them was related to restful sleep. Nevertheless, with regards to our second research question our results show that restful sleep, physical activity, and fruit and vegetable intake were associated with increased sleep quality, and that physical activity and fruit and vegetable intake were positively related to quality of life and subjective health. Sleep quality was also associated with increased quality of life and subjective health. To address our third research question, we investigated age-group differences and could show that older adults had the highest levels of being regularly physically active and consuming sufficient fruit and vegetables. With respect to sleep quality, quality of life, and subjective health, middle-aged adults yielded the lowest values. Finally, the engagement in multiple health behaviors was associated with sleep quality, quality of life, and subjective health across age groups, predominantly among middle-aged adults.

### Correlation of Main Variables

Physical activity and fruit and vegetable intake were interrelated which aligned to a previous study (Fleig et al., 2015); however, restful sleep was not interrelated with physical activity and fruit and vegetable intake which was inconsistent with a previous study (El Ansari et al., 2011). This may be due to the single item to measure sleep may not be sufficient. Some studies suggested alternative measurements like The Pittsburgh Sleep Quality Index (PSQI) (Buysse et al., 1989) may be more suitable for measuring sleep as health behavior, as PSQI includes sleep duration, sleep quality, habitual sleep efficiency, sleep disturbances, use of sleep medications, and daytime dysfunction. Restful sleep was only correlated with sleep quality, quality of life, and subjective health. This outcome is partly consistent with a recent study that showed poor sleep quality was related to poor subjective health and less restful sleep (Merrill et al., 2011; Abraham et al., 2017).

### Path Analysis of Multiple Health Behaviors and Quality of Life and Subjective Health

Based on one of the assumptions of the *CCAM*, a path model replicated the results from the earlier descriptive analyses which showed that physical activity was associated with fruit and vegetable intake but neither of them was related to restful sleep. Nonetheless, restful sleep, physical activity, and fruit and vegetable intake were all associated with increased sleep quality, which is consistent with previous studies (McKnight-Eily et al., 2011; Chan et al., 2015; Ferranti et al., 2016). The finding may indicate that other than multiple health behaviors of physical activity and fruit and vegetable intake, restful sleep should also be integrated into a healthy lifestyle, as suggested in past studies (Perry et al., 2013; Chaput and Dutil, 2016). In these studies, especially those persons who reported poor sleep had a lower likelihood to maintain their health behavior change (Hui and Grandner, 2015).

Our path models also show that physical activity and fruit and vegetable intake were associated with quality of life and subjective health. This can also be found in previous research (Södergren et al., 2012; Kwon et al., 2015). In the present study, sleep quality was related to the increased quality of life and subjective health, which is also consistent with findings in previous studies (Merrill et al., 2011; Chen et al., 2014; Abraham et al., 2017). Given the associations between these multiple health behaviors as well as quality of life and subjective health, the findings highlight that multiple health behaviors play a role in health and well-being (Geller et al., 2017), which may then also be addressed in the prevention of NCDs (Shan et al., 2015; Ferranti et al., 2016; Montag et al., 2017).

### Age Differences in Multiple Health Behaviors and Underlying Mechanisms

Health behavior change differs by age and health status (Zanjani et al., 2006), however, NCDs can affect people of all age groups (World Health Organization [WHO], 2014, 2017). This present study found significant age differences regarding the motivation to change one’s health behaviors, the actual behavior, and well-being: Middle-aged adults showed lower engagements of multiple health behavior than older adults, had poorest sleep quality, as well as the lowest quality of life and subjective health among age groups. In line with past research, middle-aged adults reported not only poor sleep quality but also yielded poor health characteristics (Haseli-Mashhadi et al., 2009; Tan et al., 2015; Rayward et al., 2017). Among age groups, most middle-aged adults in this study were working full-time and were married. Therefore, time constraints with a tight schedule might be a consequence of the multiple roles they fulfill in their daily lives, hindering them to engage in regular health behaviors (Kelly et al., 2017). Together with the decline and limitations of physical functions, adopting and maintaining healthy behaviors is exceptionally challenging for middle-aged adults. Web-based applications should be used to assist these persons in overcoming this challenge as they have proven effective among these age groups (Merrill et al., 2011; Schulz et al., 2014; Chan et al., 2015; Lippke et al., 2015; Duan et al., 2017).

In previous studies, older adults were less likely to meet the recommended level of physical activity although they were more likely to meet the recommendation of a healthy diet (Alley et al., 2017). However, in the present study, we found that older adults had more physically active and consuming sufficient fruit and vegetables than the younger groups and that the quadratic trend increased across age groups. This finding may imply that older adults were more likely to successfully engage in multiple health behaviors simultaneously compared to younger adults, which could be due to a self-selection process in which older adults were more likely to participate in this study because of their awareness of aging with health declination (Davis et al., 1994). Since the risks of morbidity and mortality caused by NCDs are high in this age group, they are likely to have higher engagement in multiple health behaviors (Shaw and Agahi, 2012; World Health Organization [WHO], 2014). Another reason could be that the reduced daily stress due to retirement. Retirement might provide more flexibility and time in contrast to those adults who work or study. However, these assumptions should be evaluated further in future research.

### Health Behaviors and Underlying Mechanisms Across Age Groups

To identify the associations of both behavioral and psychological mechanisms, the proposed path analysis model demonstrated significant age-groups differences on multiple health behaviors, sleep quality, quality of life, and subjective health.

Physical activity was not directly associated with quality of life and subjective health for most age groups, which is in contrast to previous studies (Merrill et al., 2011; Pucci et al., 2012). This may be due to most of the previous studies have adopted measurements of frequency or intensity levels of physical activity, such as the International Physical Activity Questionnaire (IPAQ) (Craig et al., 2003; Pucci et al., 2012). In the present study, we used a measure of stages of behavior change instead, which might be useful in extending beyond the frequency but also their intention to initiate and maintain the actual action of health behavior. The stages of behavior change have been considered as linear with increasing intention and action, which also has been included in analyses in previous studies (Marcus et al., 1994; Lippke et al., 2009; Plotnikoff et al., 2009; Lippke et al., 2010).

For young adults, physical activity was neither significantly associated with fruit and vegetable intake nor with quality of life and subjective health, in contrast to middle-aged and older adults. These findings are similar to previous studies that showed that these missing associations between multiple health behaviors and quality of life and subjective health among young adults may imply that multiple health behaviors are less relevant for quality of life and subjective health than among older adults (Ferranti et al., 2016; Alley et al., 2017). This may be the case because younger adults usually perceive themselves as healthier with a higher quality of life or the health outcomes they could gain from the health behaviors were less apparent. This is not the case with middle-aged and older adults, where the opposite is rather the case and where health outcomes are more prospective when they engaged in health behaviors. Indeed, older adults who showed having more engagement in multiple health behaviors yielded significant associations with increased sleep quality, quality of life, and subjective health. Especially fruit and vegetable intake was significantly associated with quality of life and subjective health among older adults, this may be consuming fruit and vegetable intake is more manageable than other health behavior such as physical activity, which highly rely on the health conditions and physical functionality.

Although causal relationships cannot be drawn in this study, the findings suggest that fruit and vegetable intake may be mediated by the relationship between physical activity and sleep quality, which suggested the multiple health behaviors may increased sleep quality. This is an important indicator for coping well with stress and improving well-being (Merrill et al., 2011; Lippke, 2014; Geller et al., 2017; Palmer and Alfano, 2017). Similarly, the findings suggest that sleep quality seems to be a potential mediator of the relationship between health behavior and quality of life and subjective health. This may bring in another possible perspective on good-quality sleep, because decreases stress levels and increased well-being may mediate the relationship between health behavior and achieving quality of life and subjective health, as suggested in the CCAM by Lippke (2014). Therefore, further mediation and moderation analyses are required to validate this assumption in the future study.

### Limitations

There are limitations of this current study which need to be considered. First, based on the cross-sectional study design, it is not possible to draw any conclusions about causality and directions among the main variables of health behaviors, sleep measures, and psychological measures. Second, a self-report measure was used to obtain reliable outcomes with a minimum of subject response bias. However, the outcomes may suffer from a lack of accuracy due to over- or underestimation of the participants’ actual health behaviors, in which a previous study suggests that people tend to overestimate their physical activity and fruit and vegetable intake (Watkinson et al., 2010). Third, due to the recruitment strategies, selection bias might have occurred since people who had a high motivation to change their health behavior may have registered themselves for participation. A fourth limitation is the unequal sample size of each age group (with middle-aged adults being the majority) which may distort the outcomes. A fifth limitation is the adoption of binary category for gender which excluded some participants from the study. Therefore, more inclusive gender options should be included in the future study. Moreover, the items of physical activity and fruit and vegetable intake were used to access the stages of change that combined both intention and action may yield different outcomes. Thus, the measurements of the health behaviors should be further investigated more indicators of health behavior change and separately as either intention or actual behavior. Lastly, such health behaviors as sleep, physical activity, and fruit and vegetable intake are complex, and the model proposed here most likely does not account for all relevant factors. For example, future studies should investigate more complex health factors such as a high-fat diet, which may affect the intensity level of physical activity on eating behavior (Beaulieu et al., 2017), as well as genetic predispositions and stable personality characteristics (Conner et al., 2017). Nonetheless, the findings suggested constructive outcomes with distinctive patterns.

### Strengths

To our knowledge, the current study seems to be one of the few studies examining restful sleep, physical activity, and fruit and vegetable intake, as well as sleep quality, quality of life, and subjective health in combination across age groups. So far, most previous studies investigated these variables separately. In the current body of literature, most previous studies investigated sleep duration and daytime sleepiness whereas in this study we investigated restful sleep as a more subjective outcome of sleep quality that might be more relevant to people’s subjective health. The application of path analysis and multi-group analysis in structural equation modeling should be mentioned as a strength because of the findings may provide more information regarding behavioral and psychological mechanisms based on the *CCAM*. And finally, we compared three age groups in contrast to previous studies that compared only young and older adults (Zilli et al., 2009; Alley et al., 2017). This study also investigated middle-aged adults as this particular age group has been suggested to be examined further in improving health outcomes with multiple health behavior change interventions (Rayward et al., 2017).

## Conclusion

Combining multiple health behaviors such as regular physical activity and healthy diet has a greater impact on health and well-being as single health behavior only (Geller et al., 2017). Notwithstanding, the concluding remark of this present study is the main findings suggested possible relationships among the multiple health behaviors and their associations with overall well-being. For example, sleep is associated with other health behaviors in living a healthy lifestyle. Altogether, a multiple health behavior approach may be effective in preventing lifestyle-related diseases and may lead to better quality of life. The findings of this present study add value to the existing literature by providing more information to health-related and medical Internet research. The findings should be considered in lifestyle management interventions to enhance health and well-being, which may be useful for public health, health care system, and policy.

Although the findings from this web-based study are applicable to all age groups, knowing the age-group differences is beneficial to take action by customizing and planning strategic interventions to reduce health risks and improve health in general. For example, the associations of multiple health behaviors with sleep quality, quality of life, and subjective health were less relevant and apparent to young adults; middle-aged adults seemed to be more inclined to achieve better sleep quality, quality of life, and subjective health if they engaged in all multiple health behaviors; and older adults were more in favor of consuming fruit and vegetables. Knowing such differences are informative in adopting the distinct approach of multiple health behavior change, since different age groups might yield noticeably different approaches in improving health.

As suggestions for future studies, sociodemographic factors, such as gender and income may yield distinct health outcomes. For instance, a study showed that insufficient sleep was more likely among females and those with lower income (Grandner et al., 2015). Moreover, the directions and causality of the relationships of the main variables should be examined further in future research, To understand more of the underlying psychological mechanisms, social-cognitive factors should be investigated further. For example, a study identified that outcome expectancy and self-efficacy were the main predictors for successful behavior change (Klusmann et al., 2016).

## Author Contributions

All authors substantially contributed to the work of this manuscript, and approved the submitted version for publication. First author, ST made significant contributions to draft this manuscript, performed the statistical analyses, and interpreted the data for the work. All co-authors significantly contributed to revise the manuscript critically, and design of the study, and organized the database.

## Conflict of Interest Statement

HdV is the scientific director of Vision2Health, a company that licenses evidence-based innovative computer-tailored health communication tools. The remaining authors declare that the research was conducted in the absence of any commercial or financial relationships that could be construed as a potential conflict of interest.

## Abbreviations

BMI: body mass index
CCAM: compensatory carry-over action model
CVDs: cardiovascular diseases
NCDs: non-communicable diseases

## References

[B1] Abraham O. Pu J. Schleiden L. J. Albert S. M. (2017). Factors contributing to poor satisfaction with sleep and healthcare seeking behavior in older adults. *Sleep Health* 3 43–48. 10.1016/j.sleh.2016.11.004 28346150 PMC10124133

[B2] Alley S. J. Duncan M. J. Schoeppe S. Rebar A. L. Vandelanotte C. (2017). 8-year trends in physical activity, nutrition, TV viewing time, smoking, alcohol and BMI: a comparison of younger and older Queensland adults. *PLoS One* 12:e0172510. 10.1371/journal.pone.0172510 28248975 PMC5332140

[B3] Andresen E. M. Malmgren J. A. Carter W. B. Patrick D. L. (1994). Screening for depression in well older adults: evaluation of a short form of the CES-D (Center for Epidemiologic Studies Depression Scale). *Am. J. Prev. Med.* 10 77–84. 10.1016/S0749-3797(18)30622-6 8037935

[B4] Araújo L. S. Wasley D. Perkins R. Atkins L. Redding E. Ginsborg J. (2017). Fit to perform: an investigation of higher education music students’ perceptions, attitudes, and behaviors toward health. *Front. Psychol.* 8:1558. 10.3389/fpsyg.2017.01558 29066983 PMC5641399

[B5] Barber L. (2014). Conceptualizations of sleep in stress theory: exciting new directions. *Stress Health* 30 431–432. 10.1002/smi.2598 25056684

[B6] Bassett E. Moore S. (2014). Neighbourhood disadvantage, network capital and restless sleep: is the association moderated by gender in urban-dwelling adults? *Soc. Sci. Med.* 108 185–193. 10.1016/j.socscimed.2014.02.029 24650740

[B7] Beaulieu K. Hopkins M. Blundell J. Finlayson G. (2017). Impact of physical activity level and dietary fat content on passive overconsumption of energy in non-obese adutlts. *Int. J. Behav. Nutr. Phys. Act.* 14:14. 10.1186/s12966-017-0473-3 28166797 PMC5294904

[B8] Buysse D. Reynolds C. Monk T. Berman S. R. Kupfer D. J. (1989). The Pittsburgh sleep quality index: a new instrument for psychiatric practice and research. *Psychiatry Res.* 28 193–213. 10.1016/0165-1781(89)90047-4 2748771

[B9] Cassidy S. Chau J. Catt M. Bauman A. Trenell M. (2016). Cross-sectional study of diet, physical activity, television viewing and sleep duration in 233110 adults from the UK Biobank; the behavioural phenotype of cardiovascular disease and type 2 diabetes. *BMJ Open* 6:e010038. 10.1136/bmjopen-2015-010038 27008686 PMC4800116

[B10] Chan T. Yen T. Fu Y. Hwang J. (2015). ClickDiary: online tracking of health behaviors and mood. *J. Med. Internet Res.* 17:e147. 10.2196/jmir.4315 26076583 PMC4526938

[B11] Chaput J. P. Dutil C. (2016). Lack of sleep as a contributor to obesity in adolescents: impacts on eating and activity behaviors. *Int. J. Behav. Nutr. Phys. Act.* 13:103. 10.1186/s12966-016-0428-0 27669980 PMC5037605

[B12] Chen X. Gelaye B. Williams M. A. (2014). Sleep characteristics and health-related quality of life among a national sample of American young adults: assessment of possible health disparities. *Qual. Life Res.* 23 613–625. 10.1007/s11136-013-0475-9 23860850 PMC4015621

[B13] Conner T. S. Thompson L. M. Knight R. L. Flett J. A. Richardson A. C. Brookie K. L. (2017). The role of personality traits in young adult fruit and vegetable consumption. *Front. Psychol.* 8:119. 10.33889/fpsyg.2017.00119 28223952 PMC5293836

[B14] Craig C. L. Marshall A. L. Sjöström M. Bauman A. E. Booth M. L. Ainsworth B. E. (2003). International physical activity questionnaire: 12-country reliability and validity. *Med. Sci. Sports Exerc.* 35 1381–1395. 10.1249/01.MSS.0000078924.61453.FB 12900694

[B15] Davis M. A. Neuhaus J. M. Moritz D. J. Lein D. Barclay J. D. Murphy S. P. (1994). Health behaviors and survival among middle aged and older men and women in the NHANES I Epidemiologic Follow-Up Study. *Prev. Med.* 23 369–376. 10.1006/pmed.1994.1051 8078859

[B16] de Vries H. van ’t Riet J. Spigt M. Metsemakers J. van den Akker M. Vermunt J. (2008). Clusters of lifestyle behaviors: results from the Dutch SMILE study. *Prev. Med.* 46 203–208. 10.1016/j.ypmed.2007.08.005 17904212

[B17] Deeks A. Lombard C. Michelmore J. Teede H. (2009). The effects of gender and age on health related behaviors. *BMC Public Health* 9:213. 10.1186/1471-2458-9-213 19563685 PMC2713232

[B18] Diener E. Pressman S. Hunter J. Delgadillo-Chase D. (2017). If, why, and when subjective well-being influences health, and future needed research. *Appl. Psychol. Health Well-Being* 9 133–167. 10.1111/aphw.12090 28707767

[B19] Duan Y. Wienert J. Hu C. Si G. Lippke S. (2017). Web-based intervention for physical activity and fruit and vegetable intake among Chinese university students: a randomized controlled trial. *J. Med. Internet Res.* 19:e106. 10.2196/jmir.7152 28396306 PMC5404143

[B20] Eaton W. W. Muntaner C. Smith C. Tien A. Ybarra M. (2004). “Center for epidemiologic studies depression scale: review and revision (CESD and CESD-R),” in *The Use of Psychological Testing for Treatment Planning and Outcomes Assessment* 3rd Edn ed. Maruish M. E. (Hillsdale, NJ: Lawrence Erlbaum) 363–377.

[B21] Eek F. Karlson B. Garde A. H. Hansen A. M. Orbæk P. (2012). Cortisol, sleep, and recovery – Some gender differences but no straight associations. *Psychoneuroendocrinology* 37 56–64. 10.1016/j.psyneuen.2011.05.003 21641118

[B22] El Ansari W. Stock C. John J. Deeny P. Phillips C. Snelgrove S. (2011). Health promoting behaviors and lifestyle characteristics of students at seven universities in the UK. *Cent. Eur. J. Public Health* 19 197–204. 22432394 10.21101/cejph.a3684

[B23] Ferranti R. Marventano S. Castellano S. Giogianni G. Nolfo F. Rametta S. (2016). Sleep quality and duration is related with diet and obesity in young adolescent living in Sicily, Southern Italy. *Sleep Sci.* 9 117–122. 10.1016/j.slsci.2016.04.003 27656277 PMC5021953

[B24] Field A. P. (2009). *Discovering Statistics Using SPSS* 3rd Edn. London: Sage.

[B25] Fleig L. Küper C. Lippke S. Schwarzer R. Wiedemann A. U. (2015). Cross-behavior associations and multiple health behavior change: a longitudinal study on physical activity and fruit and vegetables intake. *J. Health Psychol.* 20 525–534. 10.1177/1359105315574951 25903240

[B26] Frank S. Gonzalez K. Lee-Ang L. Young M. C. Tamez M. Mattei J. (2017). Diet and sleep physiology: public health and clinical implications. *Front. Neurol.* 8:393 10.3389/fneur.2017.00393 PMC5554513 28848491

[B27] Games P. Howell J. (1976). Pairwise multiple comparison procedures with unequal N’s and/or variances: a Monte Carlo Study. *J. Educ. Stat.* 1:113. 10.2307/1164979 25267786

[B28] Geller K. Lippke S. Nigg C. R. (2017). Future directions of multiple behavior change research. *J. Behav. Med.* 40 194–202. 10.1007/s10865-016-9809-8 27785652

[B29] Grandner M. A. Jackson N. J. Izci-Balserak B. Gallagher R. A. Murray-Bachmann R. Williams N. J. (2015). Social and behavioral determinants of perceived insufficient sleep. *Front. Neurol.* 6:112 10.3389/fneur.2015.00112 PMC4456880 26097464

[B30] Harvey A. G. Stinson K. Whitaker K. L. Moskovitz D. Virk H. (2008). The subjective meaning of sleep quality: a comparison of individuals with and without insomnia. *Sleep* 31 383–393. 10.1093/sleep/31.3.383 18363315 PMC2276747

[B31] Haseli-Mashhadi N. Dadd T. Pan A. Yu Z. Lin X. Franco O. H. (2009). Sleep quality in middle-aged and elderly Chinese: distribution, associated factors and associations with cardio-metabolic risk factors. *BMC Public Health* 9:130. 10.1186/1471-2458-9-130 19426521 PMC2683822

[B32] Häyrinen M. Tarkka I. (2016). Physical activity does not inevitably improve quality of life in young adults with type 1 diabetes. *Diabetes Res. Clin. Pract.* 121 99–101. 10.1016/j.diabres.2016.09.010 27690319

[B33] Hsiao Y. Y. Wu C. H. Yao G. (2014). Convergent and discriminant validity of the WHOQOL-BREF using a multitrait-multimethod approach. *Soc. Indic. Res.* 116 971–988. 10.1007/s11205-013-0313-z

[B34] Hui S. K. Grandner M. A. (2015). Associations between poor sleep quality and stages of change of multiple health behaviors among participants of employee wellness program. *Prev. Med. Rep.* 2 292–299. 10.1016/j.pmedr.2015.04.002 26046013 PMC4450439

[B35] Jaccard J. Becker M. Wood G. (1984). Pairwise multiple comparison procedures: a review. *Psychol. Bull.* 96 589–596. 10.1037/0033-2909.96.3.589

[B36] Johnson J. K. Louhivuori J. Siljander E. (2016). Comparison of well-being of older adult choir singers and the general population in Finland: a case-control study. *Music Sci.* 21 178–194. 10.1177/1029864916644486 28736492 PMC5520793

[B37] Kelly S. Martin S. Kuhn I. Cowan A. Brayne C. Lafortune L. (2017). Barriers and facilitators to the uptake and maintenance of healthy behaviours by people at mid-life: a rapid systematic review. *PLoS One* 11:e0145074. 10.1371/journal.pone.0145074 26815199 PMC4731386

[B38] Kittle K. Lee C. Waldron D. Evans M. Li Y. Dugan E. (2016). Restful sleep and driving limitations and cessation: findings from the health and retirement study. *Gerontologist* 56 571–571. 10.1093/geront/gnw162.2293

[B39] Klusmann V. Musculus L. Sproesser G. Renner B. (2016). Fulfilled emotional outcome expectancies enable successful adoption and maintenance of physical activity. *Front. Psychol.* 6:1990. 10.3389/fpsyg.2015.01990 26779095 PMC4701923

[B40] Kutner N. G. Bliwise D. L. Brogan D. Zhang R. (2001). Race and restless sleep complaint in older chronic dialysis patients and nondialysis community controls. *J. Gerontol. B Psychol. Sci. Soc. Sci.* 56 170–175. 10.1093/geronb/56.3.P170 11316835

[B41] Kwon S. C. Wyatt L. C. Kranick J. A. Islam N. S. Devia C. Horowitz C. (2015). Physical activity, fruit and vegetables intake, and health-related quality of life among older Chinese, Hispanics, and Blacks in New York City. *Am. J. Public Health* 105 S544–S552. 10.2105/AJPH.2015.302653 25905844 PMC4455524

[B42] Lachat C. Otchere S. Roberfroid D. Abdulai A. Seret F. Milesevic J. (2013). Diet and physical activity for the prevention of noncommunicable diseases in low- and middle-income countries: a systematic policy review. *PLoS Medicine* 10:e1001465. 10.1371/journal.pmed.1001465 23776415 PMC3679005

[B43] Lippke S. (2014). Modelling and supporting complex behavior change related to obesity and diabetes prevention and management with the compensatory carry-over action model. *J. Diabetes Obes.* 1 1–5. 10.15436/2376-0494.14.00925599089

[B44] Lippke S. Fleig L. Pomp S. Schwarzer R. (2010). Validity of a stage algorithm for physical activity in participants recruited from orthopedic and cardiac rehabilitation clinics. *Rehabil. Psychol.* 55 398–408. 10.1037/a0021563 21171799

[B45] Lippke S. Fleig L. Wiedemann A. U. Schwarzer R. (2015). A computerized lifestyle application to promote multiple health behaviors at the workplace: testing its behavioral and psychological effects. *J. Med. Internet Res.* 17:e225. 10.2196/jmir.4486 26429115 PMC4642394

[B46] Lippke S. Ziegelmann J. P. Schwarzer R. Velicer W. F. (2009). Validity of stage assessment in the adoption and maintenance of physical activity and fruit and vegetable consumption. *Health Psychol.* 28 183–193. 10.1037/a0012983 19290710 PMC2939463

[B47] Liu K. Daviglus M. L. Loria C. M. Colangelo L. A. Spring B. Moller A. C. (2012). Healthy lifestyle through young adulthood and the presence of low cardiovascular disease risk profile in middle age: the Coronary Artery Risk Development in (Young) Adults (CARDIA) study. *Circulation* 125 996–1004. 10.1161/CIRCULATIONAHA.111.060681 22291127 PMC3353808

[B48] Lu I. C. Yen J. M. C. Lei S. M. Cheng H. H. Wang J. D. (2011). BSRS-5 (5-item Brief Symptom Rating Scale) scores affect every aspect of quality of life measured by WHOQOL-BREF in healthy workers. *Qual. Life Res.* 20 1469–1475. 10.1007/s11136-011-9889-4 21431460 PMC3199547

[B49] Marcus B. Eaton C. Rossi J. Harlow L. (1994). Self-efficacy, decision-making, and stages of change: an integrative model of physical exercise. *J. Appl. Soc. Psychol.* 24 489–508. 10.1111/j.1559-1816.1994.tb00595.x

[B50] McKnight-Eily L. R. Eaton D. K. Lowry R. Croft J. B. Presley-Cantrell L. Perry G. S. (2011). Relationships between hours of sleep and health-risk behaviors in US adolescent students. *Prev. Med.* 53 271–273. 10.1016/j.ypmed.2011.06.020 21843548

[B51] Merrill R. M. Anderson A. Thygerson S. M. (2011). Effectiveness of a worksite wellness program on health behaviors and personal health. *J. Occup. Environ. Med.* 53 1008–1012. 10.1097/JOM.0b013e3182281145 21860328

[B52] Montag S. E. Knutson K. L. Zee P. C. Goldberger J. J. Ng J. Kim K. A. (2017). Association of sleep characteristics with cardiovascular and metabolic risk factors in a population sample: the Chicago Area Sleep Study. *Sleep Health* 3 107–112. 10.1016/j.sleh.2017.01.003 28346156 PMC5373495

[B53] Mucci N. Giorgi G. De Pasquale Ceratti S. Fiz-Pérez J. Mucci F. Arcangeli G. (2016). Anxiety, stress-related factors, and blood pressure in young adults. *Front. Psychol.* 7:1682. 10.3389/fpsyg.2016.01682 27840615 PMC5083786

[B54] Palmer C. A. Alfano C. A. (2017). Sleep and emotion regulation: an organizing, integrative review. *Sleep Med. Rev.* 31 6–16. 10.1016/j.smrv.2015.12.006 26899742

[B55] Palmiero M. Nori R. Piccardi L. (2017). Verbal and visual divergent thinking in aging. *Exp. Brain Res.* 235 1021–1029. 10.1007/s00221-016-4857-4 28032140

[B56] Perry G. S. Patil S. P. Presley-Cantrell L. R. (2013). Raising awareness of sleep as a healthy behavior. *Prev. Chronic Dis.* 10:130081. 10.5888/pcd10.130081 23928458 PMC3741412

[B57] Petry N. (2002). A comparison of young, middle-aged, and older adult treatment-seeking pathological gamblers. *Gerontologist* 42 92–99. 10.1093/geront/42.1.92 11815703

[B58] Plotnikoff R. C. Lippke S. Johnson S. T. Hotz S. B. Birkett N. J. Rossi S. R. (2009). Applying the stages of change to multiple low-fat dietary behavioral contexts. An examination of stage occupation and discontinuity. *Appetite* 53 345–353. 10.1016/j.appet.2009.07.016 19635512

[B59] Prochaska J. DiClemente C. (1983). Stages and processes of self-change of smoking: toward an integrative model of change. *J. Consult. Clin. Psychol.* 51 390–395. 10.1037//0022-006x.51.3.390 6863699

[B60] Prochaska J. J. Nigg C. R. Spring B. Velicer W. F. Prochaska J. O. (2010). The benefits and challenges of multiple health behavior change in research and in practice. *Prev. Med.* 50 26–29. 10.1016/j.ypmed.2009.11.009 19948184 PMC2813890

[B61] Pucci G. Reis R. S. Rech C. R. Hallal P. C. (2012). Quality of life and physical activity among adults: population-based study in Brazilian adults. *Qual. Life Res.* 21 1537–1543. 10.1007/s11136-011-0083-5 22362520

[B62] Rayward A. T. Duncan M. J. Brown W. J. Plotnikoff R. C. Burton N. W. (2017). A cross-sectional cluster analysis of the combined association of physical activity and sleep with sociodemographic and health characteristics in mid-aged and older adults. *Maturitas* 102 56–61. 10.1016/j.maturitas.2017.05.013 28610684

[B63] Reinwand D. Kuhlmann T. Wienert J. de Vries H. Lippke S. (2013). Designing a theory- and evidence-based tailored eHealth rehabilitation aftercare program in Germany and the Netherlands: study protocol. *BMC Public Health* 13:1081. 10.1186/1471-2458-13-1081 24245493 PMC3840618

[B64] Reinwand D. A. Crutzen R. Storm V. Wienert J. Kuhlmann T. de Vries H. (2016). Generating and predicting high quality action plans to facilitate physical activity and fruit and vegetable consumption: results from an experimental arm of a randomised controlled trial. *BMC Public Health* 16:317. 10.1186/s12889-016-2975-3 27066779 PMC4828759

[B65] Reinwand D. A. Schulz D. N. Crutzen R. Kremers S. P. de Vries H. (2015). Who follows eHealth interventions as recommended? A study of participants’ personal characteristics from the experimental arm of a randomized controlled trial. *J. Med. Internet Res.* 17:e115. 10.2196/jmir.3932 25963607 PMC4468602

[B66] Schulz D. N. Kremers S. P. Vandelanotte C. van Adrichem M. J. Schneider F. Candel M. J. (2014). Effects of a web-based tailored multiple-lifestyle intervention for adults: a two-year randomized controlled trial comparing sequential and simultaneous delivery modes. *J. Med. Internet Res.* 16:e26. 10.2196/jmir.3094 24472854 PMC3936298

[B67] Schwarzer R. (2008). Modeling health behavior change: how to predict and modify the adoption and maintenance of health behaviors. *Appl. Psychol.* 57 1–29. 10.1111/j.1464-0597.2007.00325.x

[B68] Schwarzer R. Lippke S. Luszczynska A. (2011). Mechanisms of health behavior change in persons with chronic illness or disability: the Health Action Process Approach (HAPA). *Rehabil. Psychol.* 56 161–170. 10.1037/a0024509 21767036

[B69] Shan Z. Ma H. Xie M. Yan P. Guo Y. Bao W. (2015). Sleep duration and risk of type 2 diabetes: a meta-analysis of prospective studies. *Diabetes Care* 38 529–537. 10.2337/dc14-2073 25715415

[B70] Shaw B. A. Agahi N. (2012). A prospective cohort study of health behavior profiles after age 50 and mortality risk. *BMC Public Health* 12:803. 10.1186/1471-2458-12-803 22989155 PMC3503621

[B71] Skevington S. M. Lotfy M. O’Connell K. A. Whoqol Group. (2004). The World Health Organization’s WHOQOL-BREF quality of life assessment: psychometric properties and results of the international field trial. A report from the WHOQOL group. *Qual. Life Res.* 13 299–310. 10.1023/B:QURE.0000018486.91360.00 15085902

[B72] Södergren M. McNaughton S. A. Salmon J. Ball K. Crawford D. A. (2012). Associations between fruit and vegetable intake, leisure-time physical activity, sitting time and self-rated health among older adults: cross-sectional data from the WELL study. *BMC Public Health* 12:551. 10.1186/1471-2458-12-551 22830932 PMC3487826

[B73] Storm V. Dörenkämper J. Reinwand D. A. Wienert J. de Vries H. Lippke S. (2016). Effectiveness of a web-based computer-tailored multiple-lifestyle intervention for people interested in reducing their cardiovascular risk: a randomized controlled trial. *J. Med. Internet Res.* 18:e78. 10.2196/jmir.5147 27068880 PMC4844907

[B74] Surani S. Brito V. Surani A. Ghamande S. (2015). Effect of diabetes mellitus on sleep quality. *World J. Diabetes* 6 868–873. 10.4239/wjd.v6.i6.868 26131327 PMC4478581

[B75] Tan X. Alén M. Cheng S. M. Mikkolma T. M. Tenhunen J. Lyytikäinen A. (2015). Associations of disordered sleep with body fat distribution, physical activity and diet among overweight middle-aged men. *J. Sleep Res.* 24 414–424. 10.1111/jsr.12283 25644747

[B76] Tian X. Du H. Li L. Bennett D. Gao R. Li S. (2017). China Kadoorie Biobank Study: fruit consumption and physical activity in relation to all-cause and cardiovascular mortality among 70,000 Chinese adults with pre-existing vascular disease. *PLoS One* 12:e0173054. 10.1371/journal.pone.0173054 28403155 PMC5389797

[B77] Trompenaars F. J. Masthoff E. D. van Heck G. L. Hodiamont P. P. de Vries J. (2005). Content validity, construct validity, and reliability of the WHOQOL-Bref in a population of Dutch adult psychiatric outpatients. *Qual. Life Res.* 14 151–160. 10.1007/s11136-004-0787-x 15789949

[B78] Wang J. Yang G. Wang D. Liu K. Ma Y. Liu H. (2017). Changes of peripheral lymphocyte subsets and cytokine environment during aging and deteriorating gastrointestinal tract health status. *Oncotarget* 8 60764–60777. 10.18632/oncotarget.18485 28977824 PMC5617384

[B79] Watkinson C. van Sluijs E. M. F. Sutton S. Hardeman W. Corder K. Griffin S. J. (2010). Overestimation of physical activity level is associated with lower BMI: a cross-sectional analysis. *Int. J. Behav. Nutr. Phys. Act.* 7:68. 10.1186/1479-5868-7-68 20854659 PMC2954949

[B80] WHOQOL (1993). Study protocol for the World Health Organization project to develop a quality of life assessment instrument (WHOQOL). *Qual. Life Res.* 2 153–159. 10.1007/BF00435734 8518769

[B81] World Health Organization [WHO] (2014). *Global Status Report on Noncommunicable Diseases 2014.* Switzerland: World Health Organization.

[B82] World Health Organization [WHO] (2017). *World Health Statistics 2017: Monitoring Health for SDGs.* Luxembourg: World Health Organization.

[B83] Zanjani F. A. Schaie K. W. Willis S. L. (2006). Age group and health status effects on health behavior change. *Behav. Med.* 32 36–46. 10.3200/BMED.32.2.36-46 16903613

[B84] Ziegelmann J. Lippke S. Schwarzer R. (2006). Adoption and maintenance of physical activity: planning interventions in young, middle-aged, and older adults. *Psychol. Health* 21 145–163. 10.1080/147683205001889 21985115

[B85] Zilli I. Ficca G. Salzarulo P. (2009). Factors involved in sleep satisfaction in the elderly. *Sleep Med.* 10 233–239. 10.1016/j.sleep.2008.01.004 18387848

